# In Vivo Head-to-Head Comparison of [^18^F]GTP1 with [^18^F]MK-6240 and [^18^F]PI-2620 in Alzheimer Disease

**DOI:** 10.2967/jnumed.124.268623

**Published:** 2025-02

**Authors:** Emily Olafson, Matteo Tonietto, Gregory Klein, Edmond Teng, Andrew W. Stephens, David S. Russell, Karen Pickthorn, Sandra Sanabria Bohorquez

**Affiliations:** 1 gRED, Genentech, Inc., South San Francisco, California;; 2 pRED, F. Hoffmann-La Roche, Ltd., Basel, Switzerland;; 3 Life Molecular Imaging GmbH, Berlin, Germany; and; 4 Invicro, New Haven, Connecticut

**Keywords:** tau PET, Alzheimer disease, harmonization

## Abstract

Alzheimer disease (AD) is characterized by the accumulation of tau neurofibrillary tangles that can be labeled with PET tracers. Multiple tau PET tracers have been used in clinical studies, including [^18^F]GTP1, [^18^F]PI-2620, and [^18^F]MK-6240. Standardized harmonization scales for comparing tau PET signals across tracers are currently under development and can be informed by comparisons of signals between tracers in both target and off-target regions of the brain. **Methods:** We conducted a head-to-head study comparing [^18^F]GTP1 with [^18^F]PI-2620 and [^18^F]MK-6240 in terms of dynamic range, magnitude of uptake, and correlation between tracers in participants with normal cognition and prodromal to mild AD. **Results:** [^18^F]GTP1 exhibited retention patterns that correlated with [^18^F]PI-2620 and [^18^F]MK-6240 for all Braak regions (except Braak II). Differences in tracer binding in AD target regions were relatively small, and off-target binding profiles were unique to each tracer. **Conclusion:** Our findings indicate that [^18^F]GTP1, [^18^F]PI-2620, and [^18^F]MK-6240 display similar uptake patterns in AD patients, suggesting that they detect the same tau pathology. However, the tracer-specific off-target signal distribution may impact their direct comparability, and for some use cases, tracer-specific considerations should be taken into account in the development of a standardized harmonization scale for tau PET.

## Visual Abstract

**Figure:**
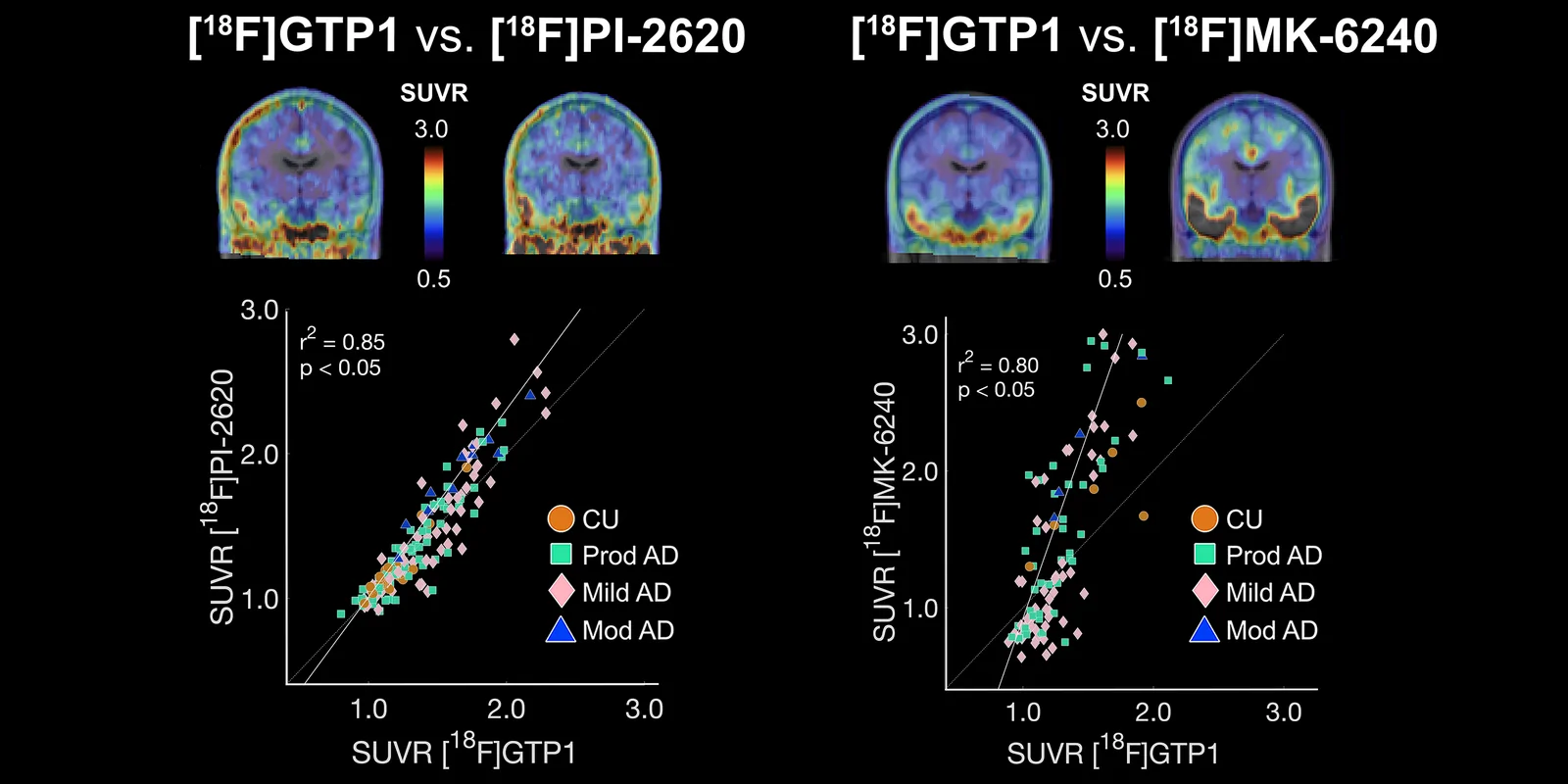

Alzheimer disease (AD) is characterized neuropathologically by the accumulation of β-amyloid plaques and tau neurofibrillary tangles. Over the past 2 decades, multiple PET tracers have been developed to quantify neurofibrillary tangles in AD. Tau PET tracers are increasingly being used in clinical trials as an enrichment biomarker to select participants at higher risk for clinical decline and who respond well to antiamyloid treatments (1) as well as a downstream pharmacodynamic biomarker to measure the effect of treatment on tau accumulation.

Multiple tau PET tracers are currently being used in clinical settings, including first-generation ligands such as [^18^F]flortaucipir (2) and second-generation ligands such as [^18^F]GTP1 (3), [^18^F]PI-2620 (4), [^18^F]MK-6240 (5,6), and [^18^F]RO948 (7). Although all tracers reliably detect AD-associated tau aggregates in vivo (3,5,8), each tracer has unique binding properties and kinetics, preventing the direct comparison of uptake across tau PET tracers and limiting studies to a single tracer.

A similar limitation was faced with amyloid PET tracers, in which uptake also cannot be directly compared. In response, the field developed the Centiloid scale (9), which converts each tracer’s uptake into a common reference scale. The Centiloid scale enables the use of multiple tracers in a single study and provides a standard unit of measurement for amyloid PET (10–12).

Recently, many single-tracer tau PET datasets have accumulated across clinical trials and publicly available sources, making tau PET harmonization imperative for comparing results across studies and conducting large-scale analyses of tau PET in AD. A novel framework for harmonizing tau PET tracers using a reference scale akin to the Centiloid has been recently proposed, called the CenTauR (13). Although cortical uptake is generally correlated across tracers (14,15), each tracer demonstrates unique patterns of an off-target signal that may influence tracer comparability. An off-target signal in extracerebral regions that impacts the cortical signal has been reported for [^18^F]MK-6240 (5,16,17), and uptake in the choroid plexus has been observed with [^18^F]GTP1 (18) and [^18^F]flortaucipir (19,20).

Head-to-head datasets, in which each subject is imaged with more than 1 tracer, enable direct comparison of tracer uptake in target and off-target regions. In recent studies, [^18^F]MK-6240 and [^18^F]flortaucipir, as well as [^18^F]RO-948 and [^18^F]flortaucipir, and [^18^F]RO-948 and [^18^F]PI-2620 have shown good linear correspondence (14,15,21), with notable exceptions due to a tracer-specific off-target signal, particularly in the choroid plexus (15). A direct comparison of [^18^F]GTP1 with [^18^F]PI-2620 and [^18^F]MK-6240, focused on characterizing the signal differences across cortical and off-target regions, is essential to understand the feasibility of harmonization efforts that might incorporate these tracers.

In this head-to-head study comparing [^18^F]GTP1 with [^18^F]PI-2620 and [^18^F]MK-6240, we assessed differences in the dynamic range, magnitude of uptake, and correlation between tracers in cortical regions, referred to as target regions, as well as differences in the magnitude of uptake and correlation between tracers in selected off-target regions.

## MATERIALS AND METHODS

### Participants

Participants were recruited from New Haven, Connecticut, and were enrolled into 2 cohorts. In cohort 1, all participants underwent a single [^18^F]PI-2620 PET scan and a single [^18^F]GTP1 PET scan in a balanced order. In cohort 2, all participants underwent a single [^18^F]GTP1 PET scan and a single [^18^F]MK-6240 PET scan in a balanced order. One patient was scanned with all 3 tracers. The second tau PET scan visit occurred within 1 to 45 d after the first tau PET imaging visit, with a target interscan interval of fewer than or exactly 14 d.

Inclusion criteria included cognitively healthy participants aged 65 to 90 y and prodromal to moderate AD participants aged 50 to 90 y. Prodromal to moderate AD participants met the National Institute on Aging and Alzheimer’s Association core clinical criteria for mild cognitive impairment due to probable AD dementia (22,23) and had a Clinical Dementia Rating of 0.5–2 at screening. β-Amyloid PET imaging confirmed the presence of β-amyloid deposition in patients with prodromal to moderate AD. All participants had a Mini-Mental Status Examination score of 10–30 inclusive. Full inclusion and exclusion criteria and recruitment strategy can be found in the supplemental materials (available at http://jnm.snmjournals.org).

The study protocol was approved by institutional review boards (Advarra) before patient recruitment and conducted in accordance with the International Conference on Harmonization E6 Guidelines for Good Clinical Practice. Each subject provided written informed consent for participation in the study before enrollment.

### PET Image Acquisition

All PET scans were performed at a central imaging center (Invicro, New Haven, CT). The tracers’ target injected doses were administered as a bolus intravenous injection, and image acquisition was performed at the predefined optimal imaging window after injection for each tracer (Table 1).

**TABLE 1.** Tracer Doses and Imaging Times

Tracer	Target injected dose (MBq)	Imaging window (min)	Scan length (min)
[^18^F]GTP1	259 (±10%)	60–90	30
[^18^F]PI-2620	185 (±10%)	45–75	30
[^18^F]MK-6240	185 (±20%)	90–110	20

All PET images were acquired on a Siemens Biograph 6 PET/CT scanner and reconstructed with an iterative reconstruction algorithm (ordered-subset expectation maximization 4 iterations, 16 subsets) and a posthoc 5-mm gaussian filter. The list-mode data were binned into 5-min-long time frames.

### MRI Processing

A full description of the MRI processing pipeline can be found in the supplemental materials. 3-Dimensional T1-weighted images were processed using FastSurfer (Deep MI Lab) (24) to obtain cortical and subcortical parcellations, including 6 Braak regions of interest (ROIs) (Supplemental Table 1; Supplemental Fig. 1) (25,26), the meta temporal region (27), and the whole cortical gray matter. Six additional ROIs typically spared by tau pathology were considered to evaluate an off-target signal: thalamus, caudate, putamen, pallidum, choroid plexus, and skull/meninges (Supplemental Fig. 2). The choroid plexus ROI was obtained using a deep-learning segmentation model (28). Finally, the skull/meninges ROI was generated from the skull probability map obtained from a CT image and intersected with the brain mask obtained from FastSurfer (Supplemental Fig. 3).

Finally, the 3-dimensional T1-weighted images of each participant were nonlinearly normalized to the Montreal Neurological Institute space. A ROI representing the inferior cerebellar cortex was generated from the SUIT cerebellum atlas (29) and masked using the gray matter mask obtained from SPM software (Wellcome Centre for Human Neuroimaging).

### Tau PET Quantitative Analysis

A full description of the tau PET analysis pipeline can be found in the supplemental materials. SUV ratio (SUVR) images were calculated using the previously derived inferior cerebellar cortex ROI as the reference region (Supplemental Fig. 1). Mean SUVRs were extracted for all the ROIs. SUVR images were then normalized to the Montreal Neurological Institute space and smoothed with a gaussian kernel with a full width at half maximum of 5 mm for voxelwise analysis.

### Statistical Analysis

Pairwise *t* test, Pearson correlation coefficient, and linear regression analyses were used to compare [^18^F]GTP1 and either [^18^F]PI-2620 or [^18^F]MK-6240 SUVR values in the different ROIs. Linear regression coefficients (slope and intercept) were computed using a total least-squares approach and expressed with [^18^F]GTP1 as the independent variable. Confidence intervals for Pearson correlation coefficients, linear regression parameters, and paired *t* tests were calculated using bootstrapping (1,000 permutations). *P* values were calculated using permutations and were corrected for multiple comparisons using the Bonferroni adjustment.

Voxelwise paired *t* tests were computed using the Statistical Nonparametric Mapping Toolbox (SnPM13.1.05; NISOx) on the smoothed SUVR images. This analysis was run using the variance smoothing option (full width at half maximum of 5 mm) and 5,000 permutations. The variance smoothing option increases the statistical power of the test by generating smoothed pseudot statistical maps (30). A significance level was set at 0.05 after familywise error rate correction.

The signal from the choroid plexus was regressed out of the signal in Braak II by fitting a linear model, using the [^18^F]GTP1 SUVR in the choroid plexus as the independent variable. The residuals from this fitted model were extracted and evaluated in subsequent analyses.

### Data Availability

For eligible studies, qualified researchers may request access to individual patient level clinical data through a data request platform. At the time of writing this request platform is Vivli (https://vivli.org/ourmember/roche/). Up-to-date details on Roche’s global policy on the sharing of clinical information and how to request access to related clinical study documents are available at https://www.roche.com/innovation/process/clinical-trials/data-sharing. Anonymized records for individual patients across more than 1 data source external to Roche cannot, and should not, be linked because of a potential increase in risk of patient reidentification.

## RESULTS

### Sample Demographics

#### Subject Characteristics

**FIGURE 1.**
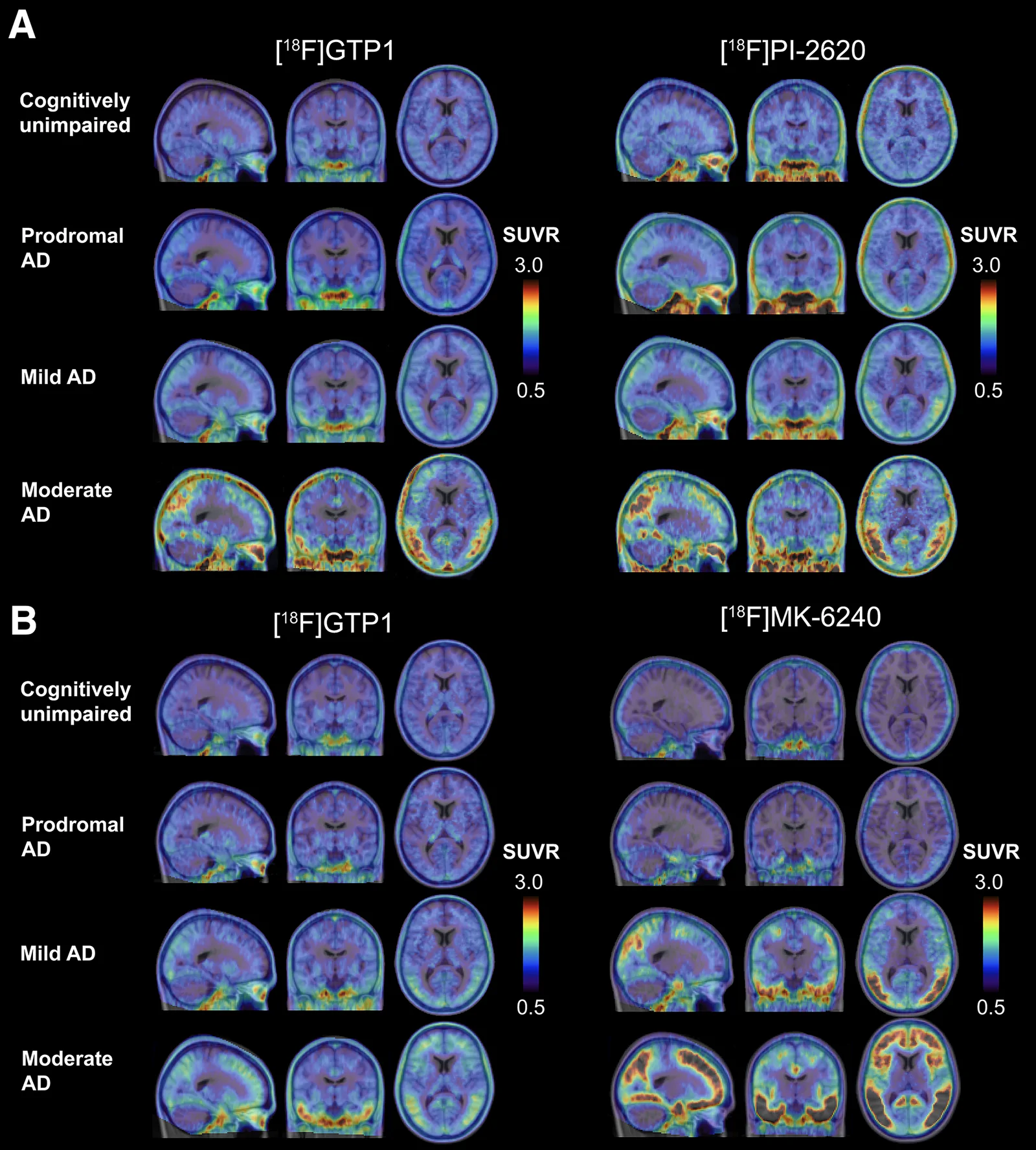
Average SUVR images from participants in cohort 1 (A), imaged with [^18^F]GTP1 and [^18^F]PI-2620, and cohort 2 (B), imaged with [^18^F]GTP1 and [^18^F]MK-6240, representing range of uptake in both cohorts. Each row contains average SUVR images from same participants.

Subject demographics and clinical characteristics are represented in Table 2. In cohort 1, 27 participants were imaged with both [^18^F]GTP1 and [^18^F]PI-2620: 5 cognitively unimpaired, 10 prodromal, 10 mild, and 2 moderate AD subjects (Fig. 1A). In cohort 2, 22 participants were imaged with both [^18^F]GTP1 and [^18^F]MK-6240: 5 cognitively unimpaired, 3 prodromal, 5 mild, and 9 moderate AD subjects (Fig. 1B). One cognitively unimpaired subject underwent a PET scan with all 3 tracers. Summary statistics for tracer dosing can be found in Table 2.

**TABLE 2.** Clinical and Demographic Characteristics of Two Cohorts

Parameter	Cohort 1: [^18^F]GTP vs. [^18^F]PI-2620					Cohort 2: [^18^F]GTP1 vs. [^18^F]MK-6240				
	Whole cohort (*n* = 27)	CU (*n* = 5)	Prodromal AD (*n* = 10)	Mild AD (*n* = 10)	Moderate AD (*n* = 2)	Whole cohort (*n* = 22)	CU (*n* = 5)	Prodromal AD (*n* = 3)	Mild AD (*n* = 5)	Moderate AD (*n* = 9)
Sex (*n*)										
Male	13	4	4	4	1	9	1	1	3	4
Female	14	1	6	6	1	13	4	2	2	5
Age (y)										
Mean (SD)	70.2 (5.3)	71.2 (0.8)	71.7 (5.4)	69.9 (5.8)	61.5 (0.7)	71.3 (6.7)	68.0 (2.5)	73.7 (4.0)	72.7 (7.7)	71.0 (8.4)
Min–max	61–80	70–72	65–80	62–77	61–62	54–82	65–71	70–78	64–82	54–82
Race (*n*)										
White	27	5	10	10	2	20	4	3	5	9
Ethnicity (*n*)										
Hispanic/Latino	2	0	1	0	1	3	0	0	1	2
Not Hispanic/Latino	25	5	9	10	1	18	5	3	4	7
Not Stated						—	—	—	—	—
Centiloid, CTL										
Mean (SD)	91.0 (41.1)	31.8 (40.4)	101.6 (34.1)	110.4 (19.2)	88.4 (33.7)	81.2 (52.3)	8.5 (23.5)	87.3 (26.6)	100.4 (20.7)	112.6 (40.7)
Min–max	−5.2–146.0	−5.2–81.1	46–146.0	77.1–140.7	64.6–112.3	−17.5–181.1	−17.5–106.1	56.8–106.1	68.6–119.1	63.2–181.1
Injected dose [^18^F]GTP1 (MBQ)										
Mean (SD)	261 (11)	258 (4)	260 (14)	265 (7)	257 (26)	260 (8)	260 (6)	255 (6)	259 (2)	259 (7)
Min–Max	228–277	254–265	228–277	257–275	238–275	247–277	247–277	248–259	257–260	247–267
Injected dose* (MBq)										
Mean (SD)	189 (11)	193 (6)	187 (12)	187 (11)	197 (3)	190 (5)	192 (3)	193 (5)	187 (10)	190 (3)
Min–max	154–199	183–198	154–196	166–198	195–199	176–197	187–194	187–197	176–196	185–194
MMSE										
Mean (SD)	25.5 (4.2)	30 (0)	26.9 (1.8)	23.8 (3.5)	16 (0)	21.8 (6.5)	29.8 (0.4)	27.6 (1.2)	20.8 (1.0)	15.9 (3.3)
Min–max	16–30	30-30	24–30	20–28	16–16	11–30	29–30	27–29	20–22	11–22
CDR-SB										
Mean (SD)	0.5 (0.3)	0 (0)	0.5 (0)	0.7 (0.3)	0.75 (0.4)	0.6 (0.6)	0 (0)	0.5 (0)	0.6 (0.2)	0.9 (0.6)
Min–max	0–1	0–0	0.5–0.5	0.5–1	0.5–1	0–2	0–0	0.5–0.5	0.5–1	0.5–2

* Injected dose of [^18^F]PI-2620 in cohort 1 and [^18^F]MK-6240 in cohort 2.

CU = cognitively unimpaired; CTL = Centiloid; MMSE = Mini-Mental State Examination; CDR-SB = Clinical Dementia Rating Sum of Boxes.

#### SUVR Comparison in Target Regions

**FIGURE 2.**
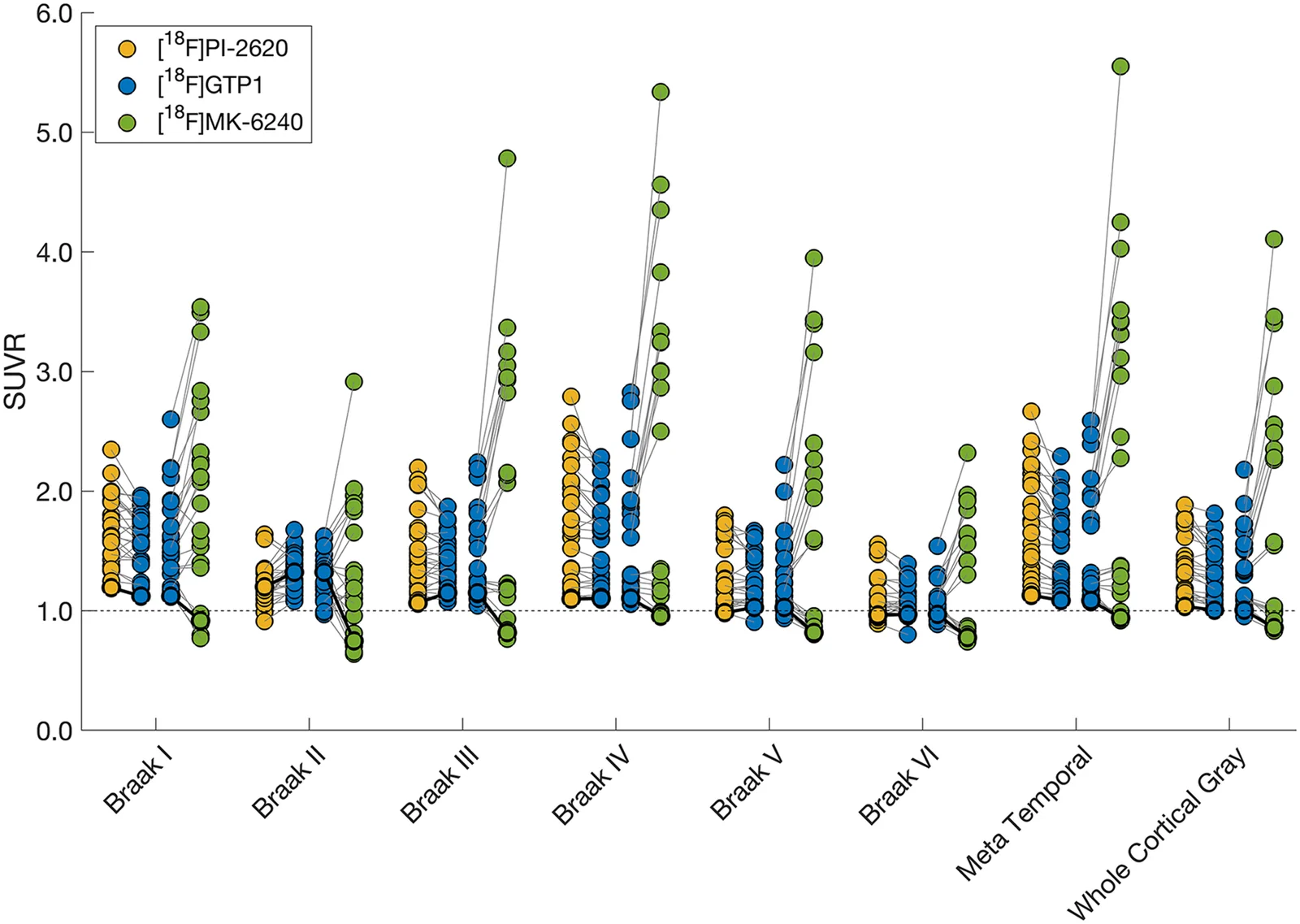
Paired plots showing [^18^F]GTP1, [^18^F]PI-2620, and [^18^F]MK-6240 SUVR values in target cortical regions. Solid lines represent 1 participant. Dashed line indicates SUVR = 1. Cognitively unimpaired participant scanned with all 3 tracers is highlighted with darker black lines.

**FIGURE 3.**
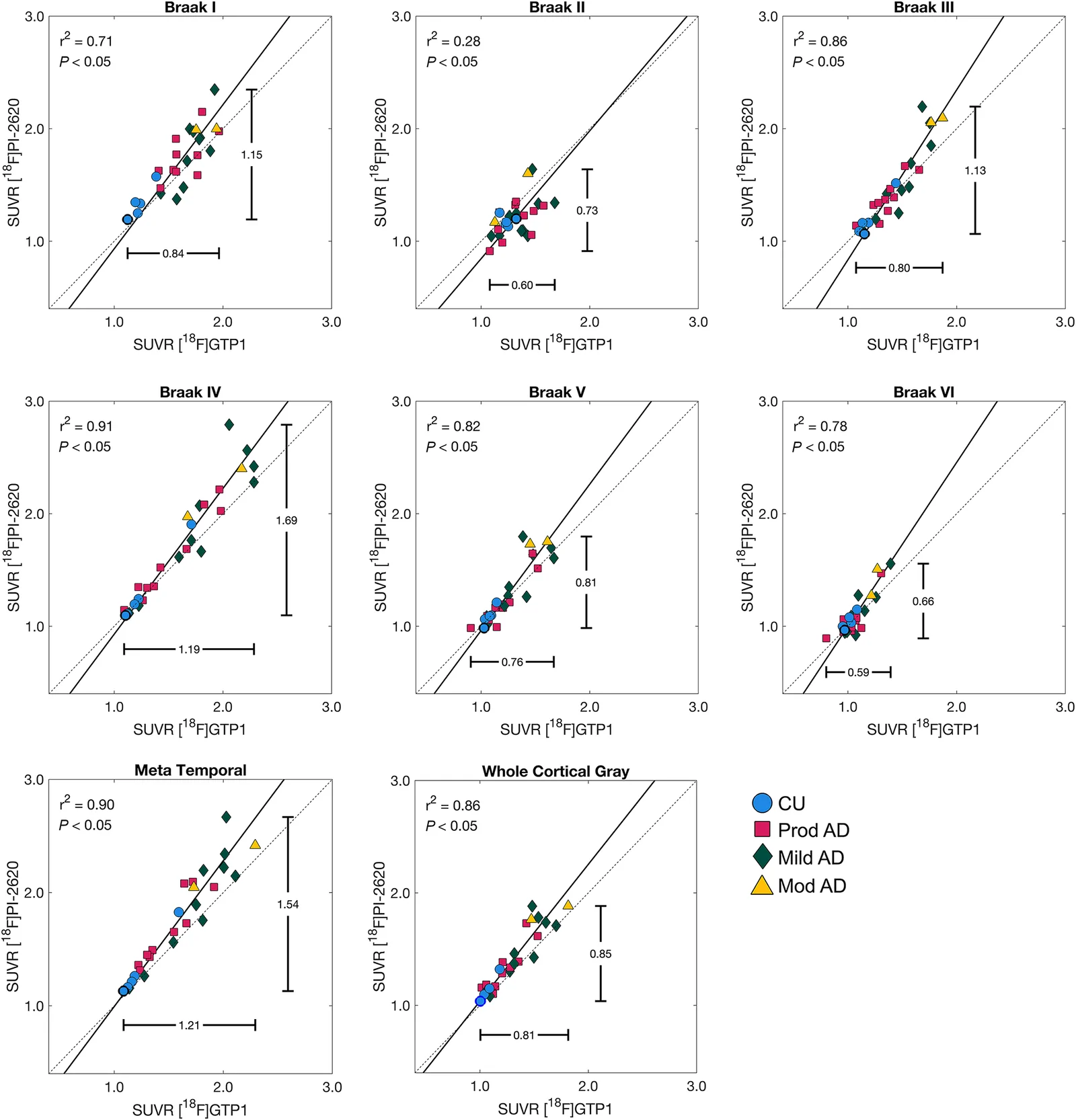
Association between [^18^F]GTP1 and [^18^F]PI-2620 SUVRs in target regions.

[^18^F]GTP1 and [^18^F]PI-2620 exhibited similar patterns of uptake in cortical ROIs (Fig. 2). [^18^F]PI-2620 had a slightly larger dynamic range than did [^18^F]GTP1 for all regions, with the exception of Braak II, as indicated by a regression slope of 1.28–1.51 (Fig. 3; Table 3). A single plot displaying all regions for each tracer can be found in Supplemental Figure 4A. The mean SUVR in Braak IV was significantly higher for [^18^F]PI-2620 than for [^18^F]GTP1 (1.72 ± 0.51 vs. 1.61 ± 0.40, *P* < 0.05), whereas [^18^F]PI-2620 had significantly lower SUVRs in Braak II compared with [^18^F]GTP1 (1.21 ± 0.17 vs. 1.32 ± 0.15, *P* < 0.05). [^18^F]GTP1 and [^18^F]PI-2620 SUVRs were strongly correlated in all on-target regions with the exception of Braak II, in which the correlation was weaker (*r*^2^ = 0.28, *P* < 0.05) (Fig. 3).

**TABLE 3.** Cohort 1 ([^18^F]GTP1 and [^18^F]PI-2620) SUVR Quantification in Target Regions

ROI	[^18^F]GTP1 SUVR	[^18^F]PI-2620 SUVR	SUVR difference ([^18^F]GTP1 − [^18^F]PI-2620)	*r*^2^ (95% CI)	Slope (95% CI)	Intercept (95% CI)
Braak I	1.61 ± 0.24	1.71 ± 0.29	−0.10 ± 0.16	0.71* (0.50–0.84)	1.28 (1.03–1.73)	−0.35 (−1.10–0.03)
Braak II	1.32 ± 0.15	1.21 ± 0.17	0.11 ± 0.16*	0.28* (0.05–0.49)	1.14 (0.51–2.33)	−0.29 (−1.88–0.52)
Braak III	1.42 ± 0.23	1.47 ± 0.34	−0.05 ± 0.15	0.86* (0.73–0.93)	1.51 (1.28–1.93)	−0.67 (−1.25 to −0.36)
Braak IV	1.61 ± 0.40	1.72 ± 0.51	−0.11 ± 0.18*	0.91* (0.80–0.96)	1.29 (1.15–1.59)	−0.36 (−0.80 to −0.16)
Braak V	1.25 ± 0.21	1.29 ± 0.27	−0.04 ± 0.12	0.82* (0.64–0.91)	1.30 (1.06–1.68)	−0.34 (−0.77 to −0.06)
Braak VI	1.07 ± 0.13	1.10 ± 0.18	−0.03 ± 0.09	0.78* (0.54–0.91)	1.46 (1.12–1.80)	−0.46 (−0.83 to −0.09)
Meta temporal	1.58 ± 0.35	1.74 ± 0.44	−0.16 ± 0.16*	0.90* (0.79–0.94)	1.29 (1.12–1.54)	−0.31 (−0.66 to −0.07)
Whole cortical gray	1.29 ± 0.23	1.39 ± 0.27	−0.10 ± 0.11*	0.86* (0.72–0.92)	1.23 (1.04–1.52)	−0.19 (−0.53–0.07)

* Indicates *P* (Bonferroni) < 0.05.

Slope of 2 indicates that for one-unit change in SUVR of [^18^F]GTP1, there is corresponding change in SUVR of 2 of [^18^F]PI-2620, on average.

Continuous data are median and range.

**FIGURE 4.**
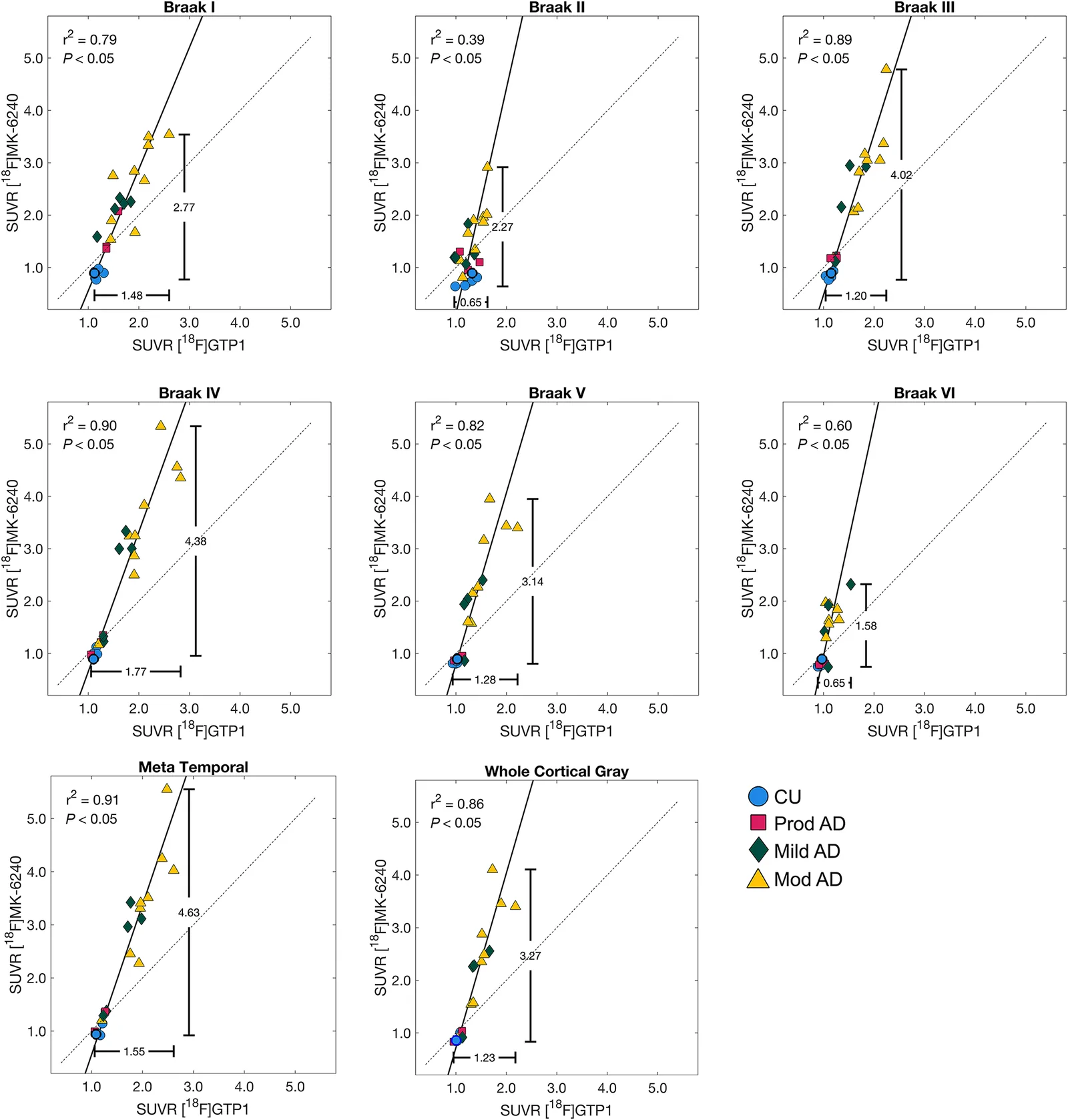
Association between [^18^F]GTP1 and [^18^F]MK-6240 SUVRs in target regions.

Patterns of uptake in the cortical ROIs were also similar for [^18^F]GTP1 and [^18^F]MK-6240 (Fig. 2). The dynamic range of SUVRs was larger for [^18^F]MK-6240 than for [^18^F]GTP1, with regression slopes ranging from 2.32 to 4.46 (Fig. 4; Table 4). A single plot displaying all regions for each tracer can be found in Supplemental Figure 4B. The mean SUVR in Braak III was significantly higher for [^18^F]MK-6240 than for [^18^F]GTP1 (1.99 ± 1.12 vs. 1.49 ± 0.38, *P* < 0.05). [^18^F]GTP1 and [^18^F]MK-6240 SUVRs were positively correlated in all target regions, with a weaker correlation observed in Braak II (*r*^2^ = 0.39, *P* < 0.05) (Fig. 4). In some cases, [^18^F]MK-6240 SUVRs were below 1, whereas [^18^F]GTP1 SUVRs were above 1.

**TABLE 4.** Cohort 2 ([^18^F]GTP1 and [^18^F]MK-6240) SUVR Quantification in Target Regions

ROI	[^18^F]GTP1 SUVR	[^18^F]MK-6240 SUVR	SUVR difference ([^18^F]GTP1 – [^18^F]MK-6240)	*r*^2^ (95% CI)	Slope (95% CI)	Intercept (95% CI)
Braak I	1.61 ± 0.41	1.97 ± 0.87	−0.36 ± 0.54	0.79* (0.46–0.91)	2.32 (1.87–2.86)	−1.77 (−2.52 to −1.01)
Braak II	1.28 ± 0.20	1.32 ± 0.57	−0.04 ± 0.48	0.39* (0.08–0.65)	4.32 (2.82–7.68)	−4.21 (−8.72 to −2.27)
Braak III	1.49 ± 0.38	1.99 ± 1.12	−0.50 ± 0.77*	0.89* (0.75–0.94)	3.07 (2.54–3.59)	−2.60 (−3.25 to −1.92)
Braak IV	1.63 ± 0.54	2.34 ± 1.39	−0.71 ± 0.89*	0.90* (0.80–0.94)	2.67 (2.21–3.28)	−2.02 (−2.83 to −1.41)
Braak V	1.28 ± 0.34	1.71 ± 1.02	−0.43 ± 0.73	0.82* (0.66–0.89)	3.28 (2.40–4.64)	−2.49 (−4.01 to −1.49)
Braak VI	1.06 ± 0.15	1.23 ± 0.53	−0.17 ± 0.42	0.60* (0.34–0.81)	4.46 (2.88–8.08)	−3.51 (−7.21 to −1.92)
Metatemporal	1.62 ± 0.49	2.31 ± 1.36	−0.69 ± 0.90*	0.91* (0.78–0.96)	2.85 (2.42–3.39)	−2.31 (−3.03 to −1.72)
Whole cortical gray	1.32 ± 0.33	1.78 ± 1.03	−0.46 ± 0.73	0.86* (0.73–0.93)	3.35 (2.51–4.36)	−2.60 (−3.85 to −1.70)

* Indicates *P* (Bonferroni) < 0.05.

Slope of 2 indicates that for one-unit change in SUVR of [^18^F]GTP1, there is corresponding change in SUVR of 2 of [^18^F]MK-6240, on average.

Continuous data are median and range.

#### SUVR Comparison in Off-Target Regions

**FIGURE 5.**
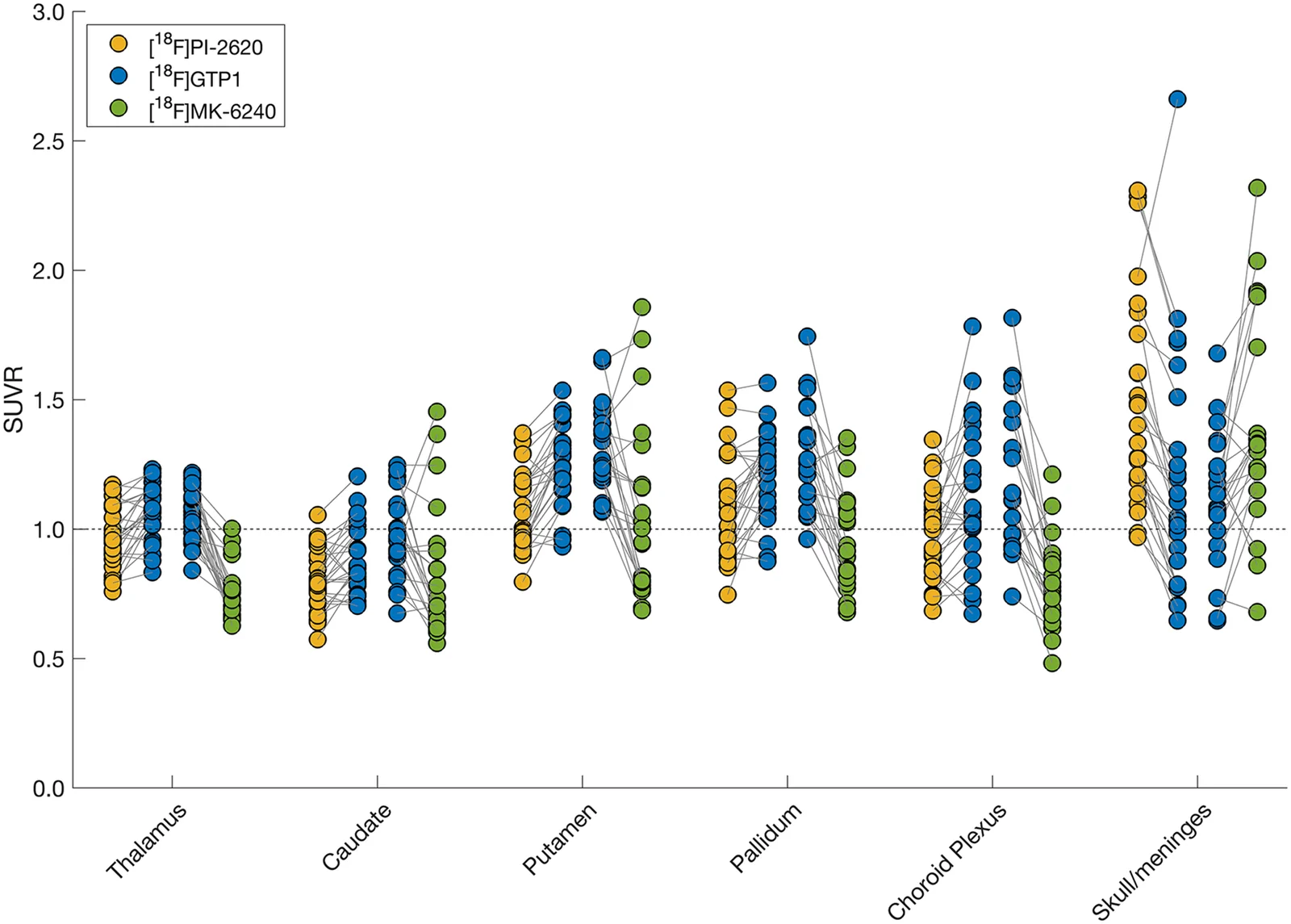
Paired plots showing [^18^F]GTP1, [^18^F]PI-2620, and [^18^F]MK-6240 SUVRs in off-target regions. Solid gray lines connect measurements from same participant. Dashed gray line is drawn at SUVR = 1.

**FIGURE 6.**
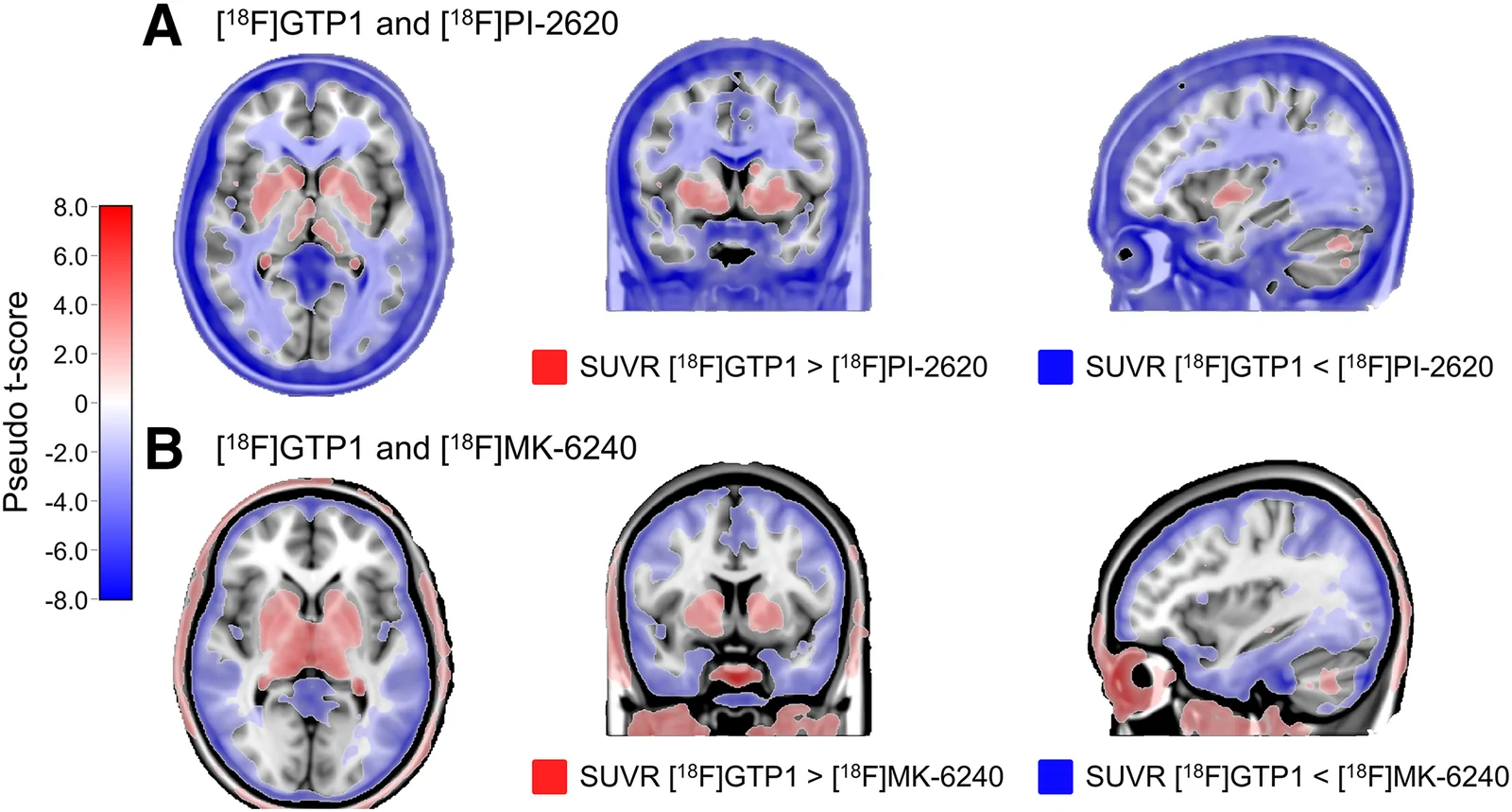
Voxelwise SUVR analysis showing voxelwise *t*-statistics from voxelwise pairwise *t*-tests overlaid on Montreal Neurological Institute T1 template. Only significant pseudo *t*-scores (family wise error rate < 0.05) are displayed.

[^18^F]GTP1 and [^18^F]PI-2620 exhibited dissimilar levels of uptake across off-target ROIs (Fig. 5). In the choroid plexus, thalamus, caudate, putamen, and pallidum, [^18^F]GTP1 SUVRs were significantly higher than [^18^F]PI-2620 SUVRs (Table 5). In the skull/meninges, [^18^F]GTP1 SUVRs were significantly lower than [^18^F]PI-2620 SUVRs (1.17 ± 0.45 vs. 1.48 ± 0.39, *P* < 0.05). Voxelwise analysis confirmed the higher SUVR of [^18^F]GTP1 in the deep gray structures and choroid plexus, and it highlighted higher SUVRs with [^18^F]PI-2620 in the white matter, skull/meninges, and base of the skull/cavernous sinus (Fig. 6A). Uptake was moderately correlated between [^18^F]GTP1 and [^18^F]PI-2620 in the skull/meninges ROI (*r*^2^ = 0.65, *P* < 0.05; Supplemental Table 2) and weakly correlated in the rest of the off-target regions (Supplemental Table 2; Supplemental Fig. 5).

**TABLE 5.** Off-Target Signal Quantification (Mean ± SD)

ROI	[^18^F]GTP1 SUVR	[^18^F]PI-2620 SUVR	SUVR difference ([^18^F]GTP1 − [^18^F]PI-2620)	[^18^F]GTP1 SUVR	[^18^F]MK-6240 SUVR	SUVR difference [^18^F] (GTP1 − [^18^F]MK-6240)
Thalamus	1.07 ± 0.11	0.96 ± 0.12	0.10 ± 0.11*	1.07 ± 0.10	0.75 ± 0.11	0.32 ± 0.15*
Caudate	0.89 ± 0.13	0.80 ± 0.12	0.08 ± 0.12*	0.97 ± 0.16	0.81 ± 0.26	0.16 ± 0.26
Putamen	1.25 ± 0.16	1.08 ± 0.15	0.18 ± 0.14*	1.30 ± 0.17	1.06 ± 0.34	0.25 ± 0.32*
Pallidum	1.21 ± 0.16	1.09 ± 0.20	0.12 ± 0.17*	1.27 ± 0.20	0.95 ± 0.19	0.32 ± 0.23*
Choroid plexus	1.13 ± 0.26	0.97 ± 0.17	0.16 ± 0.22*	1.19 ± 0.28	0.79 ± 0.17	0.40 ± 0.25*
Skull/meninges	1.17 ± 0.45	1.48 ± 0.39	−0.31 ± 0.27*	1.12 ± 0.25	1.40 ± 0.41	−0.28 ± 0.38*

* Indicates *P* (Bonferroni) < 0.05.

[^18^F]GTP1 and [^18^F]MK-6240 also exhibited different levels of uptake in off-target ROIs (Fig. 5). In the choroid plexus, thalamus, putamen, and pallidum, [^18^F]GTP1 SUVRs were significantly higher than [^18^F]MK-6240 SUVRs (Table 4). In the skull/meninges ROI, [^18^F]GTP1 SUVRs were significantly lower than [^18^F]MK-6240 SUVRs (1.12 ± 0.25 vs. 1.40 ± 0.41, *P* < 0.05). In the voxelwise analysis, [^18^F]MK-6240 exhibited higher signal in the meninges, whereas [^18^F]GTP1 had higher signal in the scalp, eyes/retina, nasal mucosa, and base of the skull/cavernous sinus (Fig. 6B). Uptake was not significantly correlated between the tracers for any off-target ROI (Supplemental Table 2; Supplemental Fig. 6).

#### Regressing out Choroid Plexus SUVR from Braak II SUVR

**FIGURE 7.**
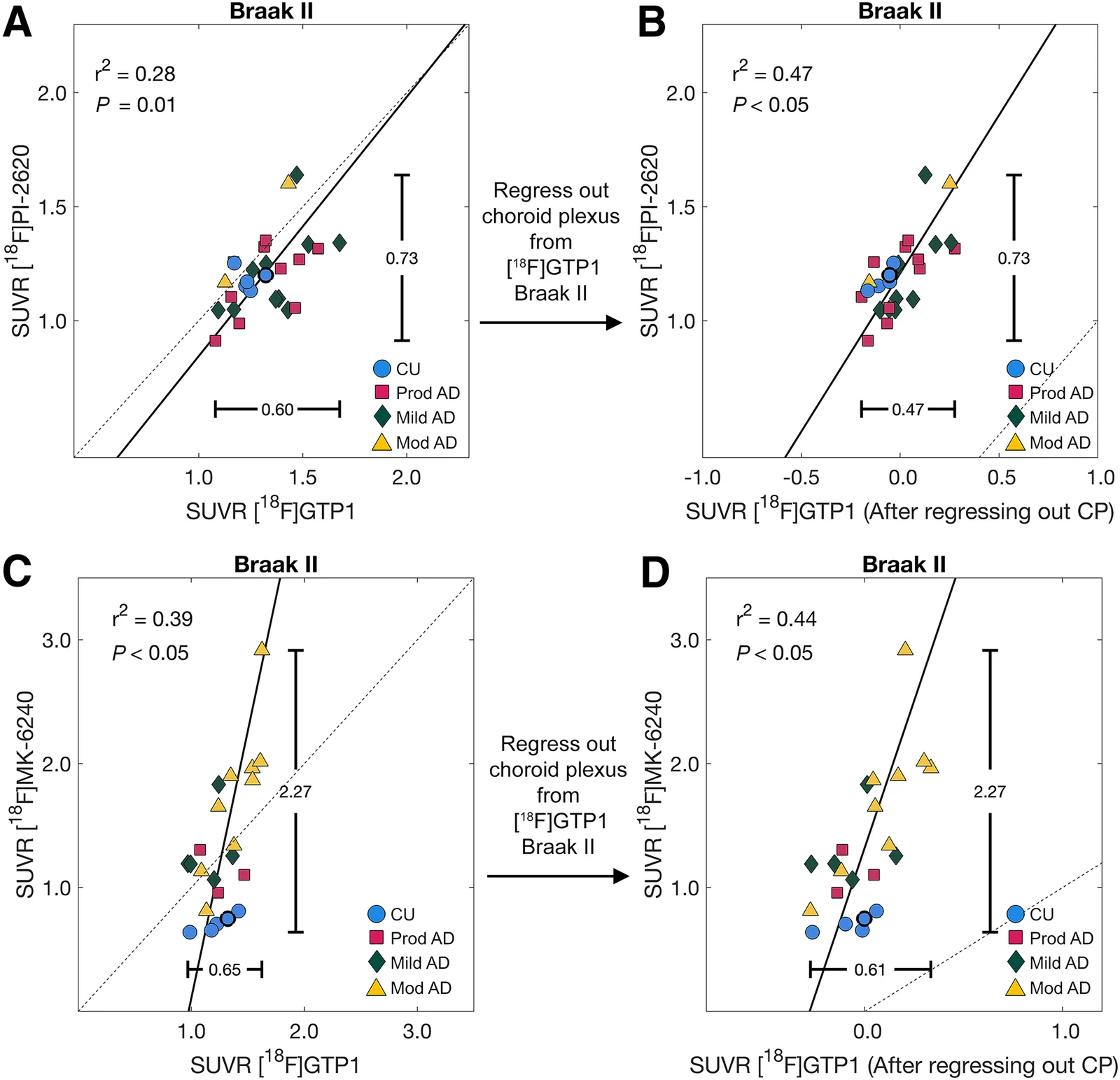
Association between [^18^F]GTP1 and [^18^F]PI-2620 (A) and [^18^F]GTP1 and [^18^F]MK-6240 in Braak II before (A and C) and after (B and D) regressing out signal from choroid plexus (CP). CU = cognitively unimpaired.

Choroid plexus SUVR was related to Braak II SUVR for [^18^F]GTP1 across both cohorts (*r*^2^ = 0.27 and 0.25 for cohort 1 and 2, respectively, *P* < 0.05) and for [^18^F]MK-6240 (*r*^2^ = 0.41, *P* < 0.05; Supplemental Fig. 7). After regressing out the choroid plexus from [^18^F]GTP1, the association between [^18^F]GTP1 and [^18^F]PI-2620 in Braak II improved from an *r*^2^ of 0.28 to an *r*^2^ of 0.47, and the association between [^18^F]GTP1 and [^18^F]MK-6240 in Braak II improved from an *r^2^* of 0.39 to an *r*^2^ of 0.44 (Fig. 7). Regressing out the choroid plexus from [^18^F]MK-6240 and [^18^F]GTP1 did not improve the association between [^18^F]GTP1 and [^18^F]MK-6240 in Braak II (original *r*^2^ = 0.39, new *r*^2^ = 0.37, data not shown).

## DISCUSSION

In this study, we directly compared uptake of [^18^F]GTP1 with [^18^F]PI-2620 and [^18^F]MK-6240, focusing on specific binding and off-target signal profiles across participants spanning the AD clinical continuum. [^18^F]GTP1 exhibited patterns of retention that were correlated with uptake of both [^18^F]PI-2620 and [^18^F]MK-6240 for all cortical regions except Braak II. Regressing out the choroid plexus signal from Braak II for [^18^F]GTP1 improved the association of [^18^F]GTP1 with [^18^F]PI-2620 and [^18^F]MK-6240. This study provides evidence that tau PET harmonization efforts that relate [^18^F]GTP1 with [^18^F]PI-2620 and [^18^F]MK-6240 are feasible.

The poor correlation between tracers in Braak II may be related to the [^18^F]GTP1 signal in the choroid plexus, which is located beside the hippocampus. The nature of this signal is unknown but may be partially related to binding to melanocytes (20) or specific binding to tau pathology that may be present in the choroid plexus (31).

The difference in the magnitude of uptake of [^18^F]MK-6240 SUVR was 2 to 3 times larger than that of [^18^F]GTP1. Similar to previous work, the distribution of uptake in Braak II with [^18^F]MK-6240 closely mapped onto diagnostic severity (15,32), suggesting that the larger dynamic range of [^18^F]MK-6240 in Braak II is disease-relevant. The wider dynamic range and larger [^18^F]MK-6240 SUVR may be related to tracer binding properties. Although [^18^F]GTP1 and [^18^F]MK-6240 bind similar targets (3,33), [^18^F]MK-6240 may bind with a higher affinity, yielding higher SUVRs in regions known to accumulate tau.

Although [^18^F]MK-6240 SUVRs were higher than [^18^F]GTP1 SUVRs at higher levels of tau burden, we observed that in some participants with low tau burden, the SUVR of [^18^F]MK-6240 was less than 1 in Braak regions, whereas [^18^F]GTP1 SUVR was greater than or equal to 1 for all participants. Similar results have been reported in previous head-to-head studies comparing [^18^F]MK-6240 to [^18^F]flortaucipir (15). Further studies are needed to investigate the nature of this discrepancy, which could be due to differences in the tracers’ binding ability to the various maturity stages of neurofibrillary tangles in AD (34). Another possibility is off-target binding of [^18^F]GTP1 (and [^18^F]flortaucipir) to a different protein in these regions or a suboptimal reference region for [^18^F]MK-6240.

[^18^F]GTP1 exhibited a higher signal in subcortical structures such as the thalamus, caudate, putamen, and pallidum compared with [^18^F]MK-6240 and [^18^F]PI-2620. The kinetics of the signal in the putamen and globus pallidus with [^18^F]GTP1 suggest that the signal in these regions does not reflect specific binding (3). Due to the location of these regions, we do not expect this signal to interfere with quantification in AD. In the skull/meninges, [^18^F]PI-2620 and [^18^F]MK-6240 displayed significantly higher binding than did [^18^F]GTP1. Melanin-containing cells in the meninges, or other sources of nondisplaceable signal, may drive the higher signal of [^18^F]PI-2620 and [^18^F]MK-6240 in this region compared with [^18^F]GTP1 (35).

Complementing recent efforts to create standardized scales for tau PET, our study suggests that tracer-specific off-target signals can degrade tracer comparability. We observed that the signal in the choroid plexus and hippocampus (Braak II) were correlated in [^18^F]GTP1 and found that regressing out the choroid plexus signal from the hippocampus improved the association of [^18^F]GTP1 with [^18^F]MK-6240 and [^18^F]PI-2620. This finding suggests that optimizing the processing for each tracer independently, before implementing a methodology for harmonization, may lead to more accurate harmonized scales. Similarly, our results suggest that a similar correction method for the [^18^F]MK-6240 and [^18^F]PI-2620 skull/meninges signal (16) could be implemented to improve harmonization with these tracers.

The limitations of this study include a relatively small sample size in both cohorts, which may contribute to less accurate head-to-head equations in AD target regions. In particular, more cognitively unimpaired amyloid-negative participants would provide insight into the nature of off-target signals and enable investigation into the low [^18^F]MK-6240 signal. Additional participants with more extensive tau pathology could also help to refine the relationship between the tracers in Braak VI and provide information on the ability of the tracers to bind neurofibrillary tangles at various stages of maturity.

## CONCLUSION

This head-to-head comparison of radiotracer uptake suggests that [^18^F]GTP1 displays retention patterns highly similar to those of [^18^F]PI-2620 and [^18^F]MK-6240 in participants with a range of Alzheimer pathology. Tracer uptake was highly correlated in target regions with the exception of the hippocampus (Braak II), but regressing out the adjacent choroid plexus signal in [^18^F]GTP1 may improve quantification. A higher off-target signal was observed in the meninges for [^18^F]PI-2620 and [^18^F]MK-6240 and in subcortical gray matter structures for [^18^F]GTP1, suggesting that off-target binding profiles of [^18^F]PI-2620 and [^18^F]MK-6240 differ from that of [^18^F]GTP1. Overall, the results support the development of a standardized harmonization scale for tau PET but suggest that optimization of the tracers’ quantification approaches are needed to account for the differences in off-target signals that can impact head-to-head relationships.

## DISCLOSURE

This work was supported by Genentech, Inc. Emily Olafson is an employee of Genentech, Inc. Sandra Sanabria Bohorquez, Matteo Tonietto, Gregory Klein, and Edmond Teng are employees of Genentech, Inc. and/or shareholders in F. Hoffmann-La Roche, Ltd. Andrew Stephens is an employee of Life Molecular Imaging GmbH. Davis Russell is an employee of Invicro. Karen Pickthorn is currently a shareholder in F. Hoffmann-La Roche, Ltd. and was an employee of Genentech, Inc. at the time of the study. No other potential conflict of interest relevant to this article was reported.

## Notes

KEY POINTS **QUESTION:** How does [^18^F]GTP1 compare with [^18^F]PI-2620 and [^18^F]MK-6240 for detecting cortical binding and off-target signal in AD? **PERTINENT FINDINGS:** [^18^F]GTP1 showed correlated retention patterns with [^18^F]PI-2620 and [^18^F]MK-6240 in most Braak regions, indicating it detects the same tau pathology. However, each tracer exhibited unique off-target binding profiles. **IMPLICATIONS FOR PATIENT CARE:** Understanding the differences in uptake patterns and off-target signal between these tracers will inform the development of standardized harmonization scales, improving the comparability of tau PET tracers and enabling multitracer analyses.
